# Transcutaneous Auricular Vagus Nerve Stimulation Modulates the Prefrontal Cortex in Chronic Insomnia Patients: fMRI Study in the First Session

**DOI:** 10.3389/fneur.2022.827749

**Published:** 2022-03-24

**Authors:** Jia-Kai He, Bao-Hui Jia, Yu Wang, Shao-Yuan Li, Bin Zhao, Zeng-Guang Zhou, Yan-Zhi Bi, Mo-Zheng Wu, Liang Li, Jin-Ling Zhang, Ji-Liang Fang, Pei-Jing Rong

**Affiliations:** ^1^Department of Physiology, Institute of Acupuncture and Moxibustion, China Academy of Chinese Medical Sciences, Beijing, China; ^2^Department of Acupuncture, China Academy of Chinese Medical Sciences Guang'anmen Hospital, Beijing, China; ^3^Department of Acupuncture, Southern Medical University, Guangzhou, China; ^4^Key Laboratory of Quantitative Remote Sensing Information Technology, Aerospace Information Research Institute, Chinese Academy of Sciences, Beijing, China; ^5^Key Laboratory of Mental Health, Institute of Psychology, Chinese Academy of Sciences, Beijing, China; ^6^Department of Radiology, China Academy of Chinese Medical Sciences Guang'anmen Hospital, Beijing, China

**Keywords:** chronic insomnia, transcutaneous auricular vagus nerve stimulation, functional magnetic resonance imaging (fMRI), biomarkers, prefrontal cortex, neuromodulation

## Abstract

**Objectives:**

Transcutaneous auricular vagus nerve stimulation (taVNS) has been reported to be effective for chronic insomnia (CI). However, the appropriate population for taVNS to treat insomnia is unclear.

**Methods:**

Total twenty-four patients with CI and eighteen health controls (HC) were recruited. Rest-state functional magnetic resonance imaging (Rs-fMRI) was performed before and after 30 min' taVNS at baseline. The activated and deactivated brain regions were revealed by different voxel-based analyses, then the seed-voxel functional connectivity analysis was calculated. In the CI group, 30 min of taVNS were applied twice daily for 4 weeks. Pittsburgh Sleep Quality Index (PSQI) and Flinders Fatigue Scale (FFS) were also assessed before and after 4 weeks of treatment in the CI group. The HC group did not receive any treatment. The correlations were estimated between the clinical scales' score and the brain changes.

**Results:**

The scores of PSQI (*p* < 0.01) and FFS (*p* < 0.05) decreased after 4 weeks in the CI group. Compared to the HC group, the first taVNS session up-regulated left dorsolateral prefrontal cortex (dlPFC) and decreased the functional connectivity (FCs) between dlPFC and bilateral medial prefrontal cortex in the CI group. The CI groups' baseline voxel wised fMRI value in the dlPFC were negatively correlated to the PSQI and the FFS score after 4 weeks treatment.

**Conclusions:**

It manifests that taVNS has a modulatory effect on the prefrontal cortex in patients with CI. The initial state of dlPFC may predict the efficacy for taVNS on CI.

## Introduction

Chronic insomnia (CI) disorder is categorized as primary or secondary, depending on whether the sleep problem is caused by another medical and mental disorder or medication substance use (1). The main treatments for CI are medications and physical therapies. Cognitive behavioral therapy (CBT), one of the most mainstream physical therapy for insomnia, was found to reduce the Functional Connectivity (FC) between the Ventral Medial Prefrontal Cortex (vmPFC) and the striatum in patients suffering from insomnia (2). Medication also affects brain activity. In healthy participants, zolpidem reduced the neural activity in occipital lobe during visual stimulation (3). Agomelatine and mirtazapine increased the FC between right Dorsolateral Prefrontal Cortex (dlPFC) and right Precuneus in Major Depression Disorder (MDD) patients with sleep disorder (4). Physical therapies have fewer side effects and therapy dependence. Guidelines of sleep disorder recommend physical therapies as the first treatment before medications (5–8).

Insomnia is also a risk factor for depression (9). It is often accompanied by mental problems (6). Colleges have to pay close attention to some potential curative effect of neuromodulations on insomnia (10), which have been widely used in the treatment of mental diseases. Deep Brain Stimulation (DBS) and Vagus Nerve Stimulation (VNS) are invasive neuromodulations, DBS was reported to have occasionally improved a patient's sleep problems in a patient with Parkinson's disease (11). Stimulating the cat's Nucleus Tractus Solitaries (NTS), the nucleus into which sensory fibers of the vagus nerve mainly project, increases the theta and beta band power of left amygdala and pre-frontal cortices. As a result, the cats performed an increase in wakefulness and a total time of rapid eye movement (REM) sleep (12). These suggest that DBS and VNS have potentially curative effect on insomnia. Acute sleep deprivation dysregulated the affective network (13–15), so it is not surprising that neuromodulations are effective on insomnia. Although many clinical trials proved their safety, surgery is still impractical for patients suffering from diseases of mild symptoms, for example, chronic insomnia (16). Transcutaneous auricular vagus nerve stimulation (taVNS) belongs to the category of neuromodulation. A clinical trial has shown the efficacy of taVNS on CI (17), but the underlying brain mechanism is still quite unclear.

Prefrontal cortex is more vulnerable to insomnia (18). The dysfunction of PFC is one of the main pathological manifestations of insomnia (19–21), neuroimaging studies reveal that sleep deprivation severely damages the PFC and reduces its ability of task execution and stimuli regulation (22). Reduced Amplitude of Low Frequency Fluctuation (ALFF) was found widely in the frontal lobe in patients with insomnia, which indicated a lower neuroexcitability. Moreover, the aberrant ALFF is related to the duration and severity of insomnia (19). Patients suffering from evening-types insomnia even have a lower metabolism and a reduced diurnal variation in PFC (23). Stimulating the peripheral branches of vagus would widely modulate the neuroexcitability through the projections from NTS to the forebrain and limbic system (24).

Our previous studies revealed that taVNS adjusts the frontal cortex, insular, PCC, and amygdala in patients with major depression disorder (25–28). The modulated brain regions were also closely related to sleep. According to the hyperarousal theory, patients with insomnia have an overexcited but low functioning cortex (18, 29), which leads to nocturnal sleep disturbances, daytime fatigue, and low work efficiency (29). In this study, the instant effects of taVNS would be explored. We hypothesize that taVNS would modulate the forebrain, especially brain regions related to emotion and cognition in patients with CI.

## Materials and Methods

### Recruitment of Participants

A total of twenty- four patients with CI were recruited. They were diagnosed according to the Fifth Edition of the Diagnostic and Statistical Manual of Mental Disorders (DSM-V, 2015). All participants were right-handed. Before the study, they were all informed of the study protocol and volunteered to participate in the study. Patients with fMRI contraindications, severe organic or mental diseases were excluded. Patients would voluntarily quit the ongoing therapies including sleeping pills for at least 2 weeks. Healthy controls (HC) were recruited, at the same time, they were matched with patients in gender, age, and education (see Table 1). All participants declaimed to have taken any sleep-aid drugs or psychotropic drugs. Both the CI group and the HC group received the same clinical assessment, a session of taVNS treatment, and fMRI scans at baseline. After that the CI group received 4 weeks' of taVNS treatment while the HC group did not receive any treatment.

**Table 1:** Sample characteristics of the participants.

**Items**	**CI (***N*** = 20)**	**HC (***N*** = 28)**	***Z*** **/χ^2^**	***p*** **-value**
Age (year)	42.50 ± 15.42	43.5 ± 11.23	−0.278	0.781
Sex (M/F)	8/12	6/12	0.181	0.671
Education (year)	12.20 ± 4.62	12.83 ± 6.24	−0.179	0.858

*Z, Wilcoxon rank testing; χ^2^, chi-square testing. CI, chronic insomnia; HC, healthy control*.

### Ethical Review and Registration

The study was reviewed by the Ethics Committee of Institute of Acupuncture and Moxibustion under China Academy of Chinese Medical Sciences (CACMS) and registered at the Chinese Clinical Trial Registry (NO. ChiCTR-15007374).

### Transcutaneous Auricular Vagus Nerve Stimulation

**Figure 1:**
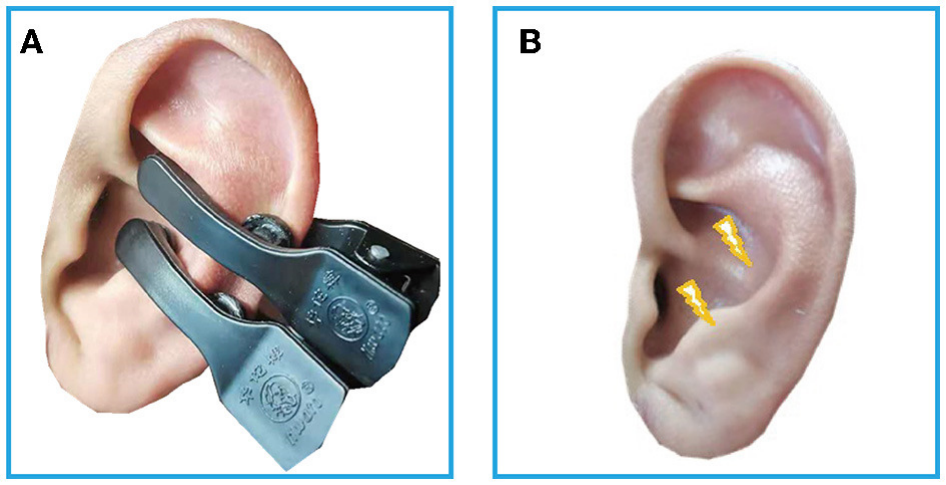
**(A)** The electrodes were attached to the surface of cymba conchae. **(B)** The stimulating place of taVNS. RS-fMRI, rest-state functional Magnetic Resonance Imaging; taVNS, Transcutaneous Auricular Vagus Nerve Stimulation.

The electro-acupuncture stimulator (SDZ-IIB, Hwato brand, made in Su zhou, China) was attached to the bilateral cymba conchae through electrodes on the skin surface (see Figure 1). Parameters were set according to previous studies of taVNS (17, 27): Dilatational wave of 4/20 Hz and pulse width of 0.2 ms ± 30%. Current intensity was adjusted according to each patient's subjective feeling. Each taVNS session lasted for 30 min, twice a day for 4 weeks, which is recommended by guidelines for short-term medications of insomnia (7, 8).

### Clinical Assessments

**Figure 2:**
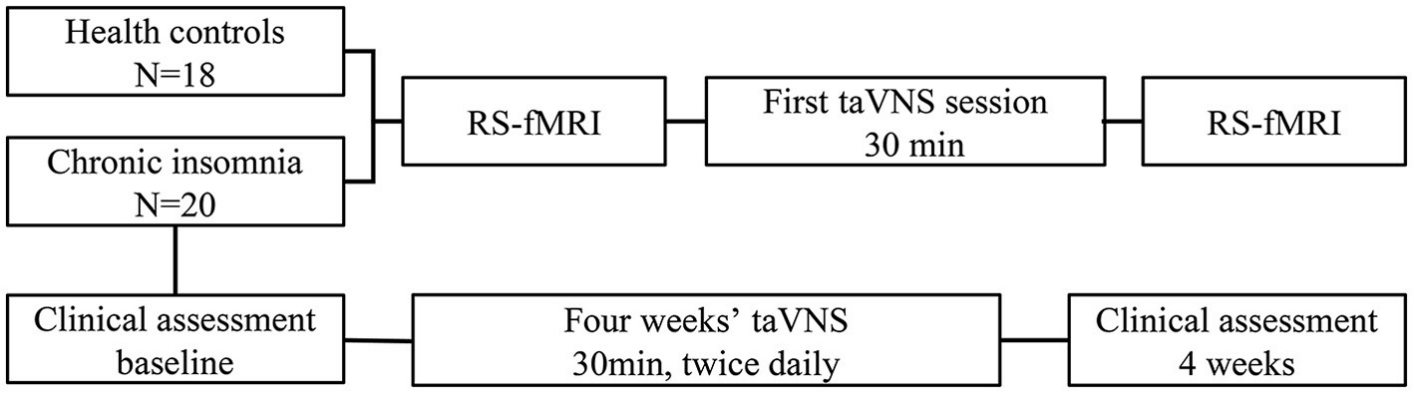
Changes of fMRI induced by instant taVNS were measured before and after the first treatment on the two groups. Clinical scales for patients were assessed before and after the 4 weeks' taVNS treatment.

All participants accepted Pittsburgh Sleep Quality Index (PSQI) and Flinders Fatigue Scale (FFS) before and after the 4 weeks' of treatment. To exclude the risk of depressive or anxiety symptoms, which may independently affect imaging findings, we used Hamilton Rating Scale for Depression (HAMD) and Hamilton Anxiety Rating Scale (HAMA) to estimate the mental status of all the participants. Before and after the taVNS treatment, the patient would be excluded with a total score of HAMD or HAMA >7. The process of this study is shown in Figure 2. In addition, we screened all patients' T2-weighted images and structural images to ruled out most of the serious metabolic or immune-related neuropsychiatric diseases, cerebrovascular diseases, inflammatory diseases of central never system, and intracranial tumors.

### MRI Data Acquisition

Rest-state functional magnetic resonance imaging (Rs-fMRI) were performed before and after the first 30 min' taVNS session. Participants were told to keep their eyes closed and not fall asleep during the scan. The fMRI data was acquired by Siemens 3.0T Skyra equipment (Siemens; Munich, Germany). The scanning parameters were as follows. In functional images, the blood oxygen level-dependent gradient Echo Planar Imaging (EPI) sequence was used. One hundred and forty four volumes lasted 6 min 10 s, repeat time/echo time: 2,500/30 ms, flip angle = 90 degrees, scanning field of view: 240 mm × 240 mm, matrix: 64 × 64, number of layers: 43, layer thickness/spacing: 3.0/1.0 mm. In high-definition structure image, three-dimensional magnetization was used to prepare fast gradient echo sequence, repeat time/echo time: 2,500/2.98 ms, flip angle: 7 degree, field of view: 256 mm × 256 mm, matrix: 64 × 64, number of layers: 48; Layer thickness/spacing: 1.0/1.0 mm.

### FMRI Data Preprocessing

DPABI (http://rfmri.org/DPABI) software (30), a SPM-based functional MRI preprocessing pipeline, was used for data preprocessing. The preprocessing steps were as follows. Convert DICOM file into NIFTI. Remove the first 10 time points. The remaining 134 volumes were slice-time corrected and realigned according to Friston 24-parameter model. The nuisance signals (including linear trend, head-motion, signals of cerebrospinal fluid, and white matter) were regressed out from the data (31). Then the functional images were co-registered to the T1-weighted structural images, which were segmented through Voxel Based Morphometry (VBM). Derived images were normalized to Montreal Neurological Institute (MNI) space according to transformation parameters estimated by VBM.

The limitations of the signal-to-noise ratio and disputes in sampling and preprocessing strategies for fMRI data, the existing voxel based analysis studies are sometimes contradictory. To get a better presentation of the short- time intervention, we employed ALFF, fALFF, and ReHo to reveal the reproductive results.

### FMRI Data Processing

The ALFF and fALFF were calculated from the normalized images after smoothing (6 mm Gaussian kernel full width at half maximum smooth nucleus) to the MNI space. ALFF value was calculated as the average square root of the power spectrum range of 0.01–0.1 Hz and converted to a frequency domain through the fast Fourier transform process. FALFF value is the ratio of power in the specific frequency band of the whole detected frequency range. The ALFF and fALFF maps were also transferred to mean ALFF and fALFF maps by subtraction of the global mean value. The mean ALFF and fALFF values were converted to Z-distribution for standardization. Then we got the zALFF and zfALFF map.

Regional homogeneity (ReHo) is calculated by voxel based on Kendall's coefficient of concordance (KCC) for the time series of a given voxel with its nearest neighbors (32). ReHo maps was calculated through the unsmoothed and filtered (0.01–0.1 Hz) images to remove physiological signals such as heartbeat and respiration. Then ReHo maps were taken to mean ReHo maps by subtraction of the mean voxel wise ReHo in the entire brain and standardized into Z-value (zReHo Maps). Calculated zReHo maps were smoothed to MNI space with 6 mm Gaussian kernel full width at half maximum smooth nucleus at last.

FC is the Pearson's correlations of the temporal fMRI signals between a Region of Interest (ROI) and all brain. Activated or deactivated regions found by the above voxel based analyses would be used as the Region of Interest (ROI) for seed to voxel FC analysis. FC were computed by voxel in the normalized image after smoothing to Montreal Neurological Institute (MNI) space (6 mm Gaussian kernel full width at half maximum smooth nucleus). All images were band-pass filtered (0.01–0.1 Hz) before FC was computed. Pearson's correlation coefficients were transformed into normally distributed scores according to the Fisher's R- to -Z transformation.

### Statistics

In SPSS 25 (SPSS Inc., Chicago, IL, USA), two sample *T*-test and χ^2^ tests were applied to compare the baseline characteristics between the CI and HC group. Paired *T*-test was applied to compare within group changes of PSQI and FFS scores in CI group.

For the fMRI images, the between group differences were performed with independent two sample *T*-test, with an uncorrected *p*-value < 0.05. Paired *T*-tests were performed to determine the within-group differences in the group, before and after the first taVNS session. For the within group comparisons, multiple comparison corrections were performance in Gaussian random field correction (GRF), combined voxel wise *p-*value < 0.001 with cluster *p-*value < 0.05 (two tailed). To clarify the behavioral associations of ALFF, fALFF, ReHo, and FC, we performed *Pearson* correlation analyses between the fMRI values and clinical scales in SPSS 25, controlling for age, sex, and education.

## Results

### taVNS Improved PSQI and FFS Scores

Out of 24 patients, two were excluded, one because of stroke history found by structural images and the other because the patient was diagnosed with bipolar disorder. Another two patients have withdrawn from the study. At last, twenty patients completed the 4 weeks' of taVNS treatment as well as the two fMRI scan sessions. The mean duration of insomnia was 95.2 months. Both PSQI (*N* = 20, *p* < 0.01 95%*CI*) and FFS (*N* = 20, *p* < 0.05, 95%CI) improved after the 4 weeks' taVNS treatment (see Table 2).

**Table 2:** Improvement of PSQI and FFS after 4-weeks taVNS treatment ($\bar{x}$ ± s).

**Items**	**Baseline**	**After treatment**	***Z***	***p*** **-value**
PSQI (*N* = 20)	12.7 ± 3.715	9.75 ± 4.278^†^	3.337	0.003
FFS (*N* = 20)	14.5 ± 5.92	11.5 ± 4.136*	2.860	0.010

* *p < 0.05*;

† *p < 0.01; Z, Wilcoxon rank testing; PSQI, Pittsburgh Sleep Quality Index; FFS, Flinders Fatigue Scale; Change at week 4 to baseline mean (95% CI)*.

### First taVNS Session Activated the Similar Location in Left dlPFC and Adjusted Its FC With PFC

**Figure 3:**
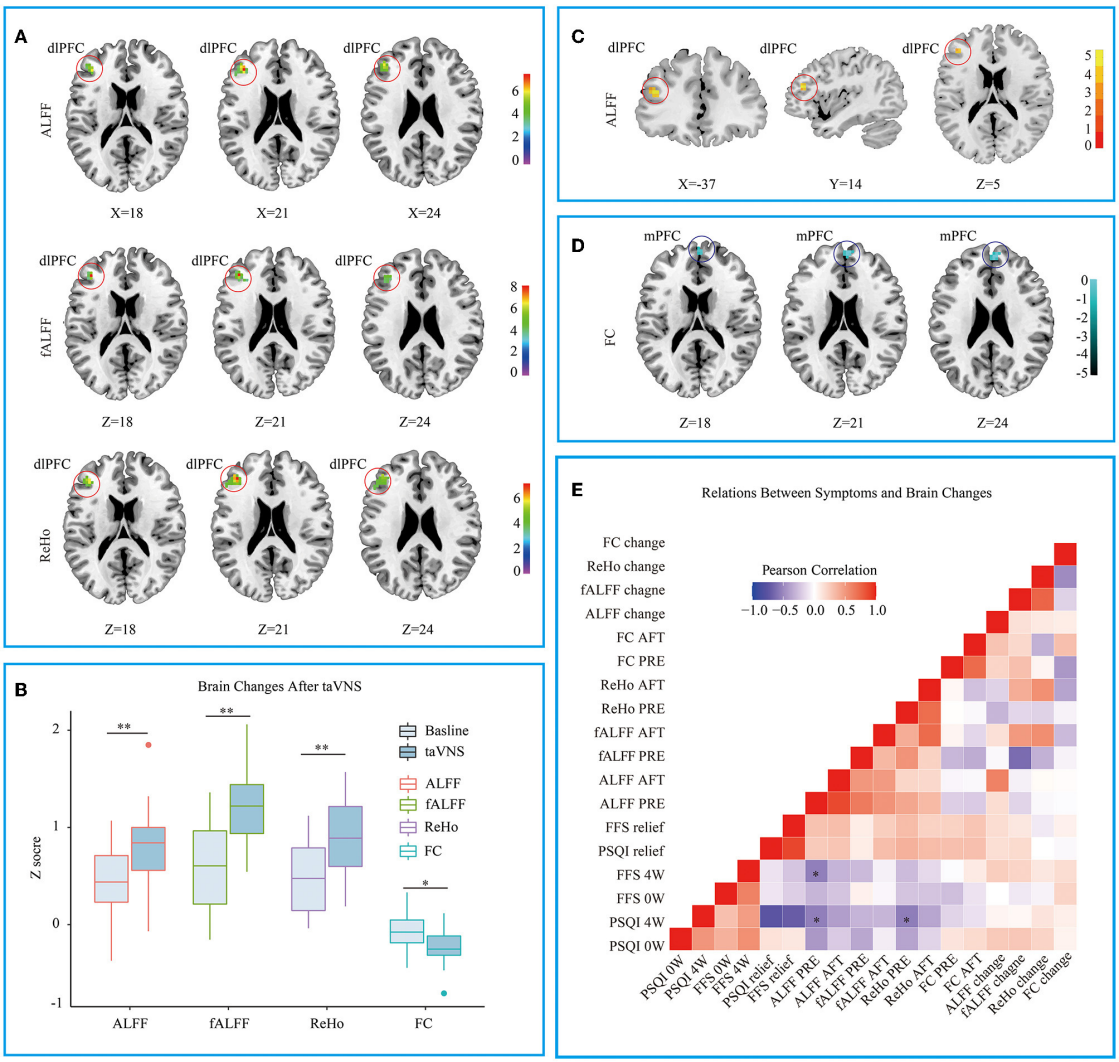
**(A)** Different voxel based analyses showed similar up- regulated area in left dorsolateral prefrontal cortex. **(B)** Changes of different voxel based analyses before and after taVNS. **(C)** ALFF analysis showed taVNS aroused higher activation in the CI group than in the HC group. **(D)** The FC between the up- regulated dlPFC and bilateral dorsomedial prefrontal cortex decreased after taVNS. **(E)** Correlations between the clinical scales' scores and the fMRI values. PSQI, Pittsburgh Sleep Quality Index; FFS, Flinders Fatigue Scale; ALFF, amplitude of low frequency fluctuation; fALFF, Fractional ALFF; ReHo, Regional homogeneity; FC, Functional connectivity; 0w, before taVNS treatment; 4w, after 4 week taVNS treatment; Relief Rate, The remission rate was defined as the difference in scale scores before and after treatment divided by the scale scores before treatment. ^*^p < 0.05; ^**^p < 0.01.

The CI group showed lower ALFF and fALFF in dlPFC and higher ReHo in Precuneus when compared to HC group (see Supplementary Material 1), which is similar to previous studies (19, 33). Three different voxel based analyses showed consistent results. Namely, the first taVNS session up- regulated left dlPFC in the CI group (see Figures 3A,B and Table 3). ALFF analysis showed the activation aroused by taVNS was higher in CI group than in the HC group (see Figure 3C). Then the activated dlPFC found by the ALFF, fALFF, and ReHo were merged as one ROI. The following seed to voxel FC analysis revealed decreased FC between dlPFC and bilateral dormedial prefrontal cortex (dmPFC) (see Figures 3B,D).

**Table 3:** Brain changes after the first taVNS session (*N* = 20).

**Items**	**Brain regions (AAL)**	**BA**	**Number of voxels**	**MNI coordinates(mm)**			**Peak intensity**
				**X**	**Y**	**Z**	
ALFF	Frontal_Mid_L Frontal_Inf_Tri_L	10/46	37	−39	36	21	7.791
fALFF	Frontal_Mid_L Frontal_Inf_Tri_L	10/46	26	−39	39	21	8.305
ReHo	Frontal_Mid_L Frontal_Inf_ Tri_L	10/45/46	71	−39	39	21	7.364
FC	Frontal_Sup_ Medial_R Frontal_Sup_ Medial_L	9/10	28	3	57	21	−5.143

*ALFF, Low frequency fluctuation in the left dorsolateral prefrontal lobe; fALFF, Fractional amplitude of low frequency fluctuation in the left dorsolateral prefrontal lobe; ReHo, Regional homogeneity in the left dorsolateral prefrontal lobe; FC, Functional connectivity between left dorsolateral prefrontal and medial prefrontal lobe; AAL, Anatomical Automatic Labeling; MNI, Montreal Neurological Institute; BA, Brodmann area*.

### A Lower ALFF or ReHo Value in dlPFC Before the First Session Correlating With the Higher PSQI Score After 4 Weeks' of Treatment

When the correlations were examined between the clinical scales' scores and the fMRI values, several significant results were defined. At baseline, ALFF values in dlPFC were negatively correlated with the patients' PSQI (*R* = −0.536, *p* < 0.01) and FFS (*R* = −0.537, *p* < 0.05) score after 4 weeks' of treatment. The baseline ReHo values in dlPFC were also negatively correlated with the after-treatment PSQI (*R* = −0.545, *p* < 0.05) (see Figure 3E).

## Discussion

Our current study revealed that taVNS improved the CI symptoms. In the first session, the taVNS up- regulated the left dlPFC and reduced its FC with bilateral dmPFC. The baseline ReHo and ALFF values in the left dlPFC were correlated with the PSQI or FFS scores after 4 weeks' of treatment.

### dlPFC Is a Potential Targeting Brain Region of taVNS Treatment on CI

According to the hyperarousal theory, the Ascending Reticular Activating System (ARAS) promotes the soberness of human brain. Patients with CI have higher FC between the thalamus and dlPFC, when compared to good sleepers. As a result, some brain regions reduce their activity to compensate for the bottom-up arousal effects originating from ARAS. For example, dlPFC show a decreased ALFF in insomniacs (21), which is similar to what we have found. DlPFC is a core region of cognitive control network (CCN) (34–37), that is why insomniacs have lower working efficiency and they are vulnerable to fatigue despite of their overexcited global status.

DlPFC is actually the most common stimulating target of transcranial magnetic stimulation (TMS). TMS on dlPFC can reduce the heart rate, the connection between the vagus and PFC is the anatomical basis of these phenomena (38). Unlike TMS, taVNS activates dlPFC indirectly. The connection offers a potent answer to what we have observed. FC Maps between dlPFC and subgenual cingulate has becoming a promising method for navigating TMS in treating depression (39). Interestingly, our study also found that patients whose initial state of dlPFC was low functioning would have a higher PSQI and FFS after 4 weeks of treatment. However, the limited sample size failed to reveal any correlation between the remission rate and ALFF values or ReHo values. A fMRI study reveals CBT increase the fALFF values in dlPFC and decrease the fALFF values in dmPFC in patient with major depression disorder (40). This phenomenon indicates increased nervous excitability in the dlPFC, which is similar to what we have found after the first session of taVNS treatment. We speculate that taVNS and CBT may share a similar brain effect on the dlPFC.

### taVNS Lowered the CCN's Monitoring to Default Mode Network

Patients with CI have an abnormal FC between default mode network (DMN) and the additional brain regions when compared with good sleepers (9, 41), which aggravate the hyperarousal status of the brain. Increased ALFF values are found in brain regions related to sensation and attention (19). That is why patients with insomnia are more sensitive to external stimuli and are easier to be awakened. The FC within DMN, especially between the prefrontal lobe and the posterior DMN, decreases when we fall asleep (19, 41). The dysfunctional DMN also leads to the abnormal FCs within DMN, which impairs both the sleep structure and working memory (33, 42). DmPFC is one of the most prominent brain regions of the abnormal frontal DMN (41, 43). Study has also confirmed that dmPFC is the key area for maintaining sleep (43). Patients with CI would pay excessive attention to sleep quality, which would aggravates frustration (44). This is because mPFC is connected with the hippocampus, amygdala, nucleus accumbens, and hypothalamus. They manage reward circuit and emotions (34, 45).

Long-term sleep deprivation leads to a decompensated Salient Network (SN). CCN should allocate more resources to compensate the loosed ability of SN to modulate the aberrant DMN. The current study found that taVNS decreased the FC between left dlPFC and bilateral dmPFC, which is opposite to the pathological changes of the patients with insomnia (2, 46), indicating that CCN has lowered its monitoring to DMN, and the excessive consumption of CCN reduced. We speculate that taVNS would alleviate the symptoms of low efficiency and fatigue in patients with CI.

Electroencephalogram (EEG) has a better time resolution than fMRI. Many neuromodulations use EEG as a brain-machine interface to improve stimulating parameters. Our study found the cortex is the most outstanding brain region affected by instant taVNS. It's easier to get stable EEG signals of the cortex. Using EEG to explore biomarkers of a certain neuromodulation would be of higher translational value than fMRI.

### Limitations

First, there was no obvious decrease in PSQI in the current study, which may be due to the fact that we only recruited patients with mild primary insomnia to ensure the consistency of the basic state. Second, while our study revealed that the initial status of PFC in patients with insomnia was related to curative effect, no difference were found between the changes of dlPFC and the changes of the patients' clinical scores. This may be due to the limited sample size. Transient taVNS cannot completely explain the efficacy of mechanism of taVNS. The positive results we found need a longer observation to get more convincing results. Third, a placebo control group is indeed the best designed control groups of this study. At last, the sample size was too small and we only studied the EPI sequence. Despite of the limitations, we intend to provide the potential predicting imaging biomarkers for the suitable patients who are sensitive to taVNS.

## Conclusions

In this study, we found a short time taVNS aroused the left PFC in patients with insomnia. The changes of PFC could be replicated through different voxel-based analyses. The projection from NTS to forebrain might be the anatomical basis of our findings.

## Data Availability Statement

The raw data supporting the conclusions of this article will be made available by the authors, without undue reservation.

## Ethics Statement

The studies involving human participants were reviewed and approved by Ethics Committee of Institute of Acupuncture and Moxibustion under China Academy of Chinese Medical Sciences. The patients/participants provided their written informed consent to participate in this study. Written informed consent was obtained from the individual(s) for the publication of any potentially identifiable images or data included in this article.

## Author Contributions

This article was written mainly by J-KH and B-HJ. The research scheme was designed by P-JR. Patients were recruited and assessed by LL and J-LZ. FMRI data were collected by BZ and were preprocessed by J-KH. Statistics and mapping were assisted by J-KH, Z-GZ, and Y-ZB. S-YL, YW, J-LF, and P-JR reviewed the article. Text correction was done by M-ZW. All authors contributed to the article and approved the submitted version.

## Funding

This study was supported by the National Key Research and Development Program (No. 2018YFC1705800) and the National Natural Science Foundation (Nos. 81473780 and 81774433).

## Conflict of Interest

The authors declare that the research was conducted in the absence of any commercial or financial relationships that could be construed as a potential conflict of interest.

## Publisher's Note

All claims expressed in this article are solely those of the authors and do not necessarily represent those of their affiliated organizations, or those of the publisher, the editors and the reviewers. Any product that may be evaluated in this article, or claim that may be made by its manufacturer, is not guaranteed or endorsed by the publisher.
