# Supplementary material for: Transcutaneous Auricular Vagus Nerve Stimulation Modulates the Prefrontal Cortex in Chronic Insomnia Patients: fMRI Study in the First Session
Source: Front Neurol. 2022 Mar 24;13:827749. doi: 10.3389/fneur.2022.827749 (PMC8987020; doi:10.3389/fneur.2022.827749)
Supplement: Supplementary file 1 [file Table_1.DOCX]

Supplementary Material


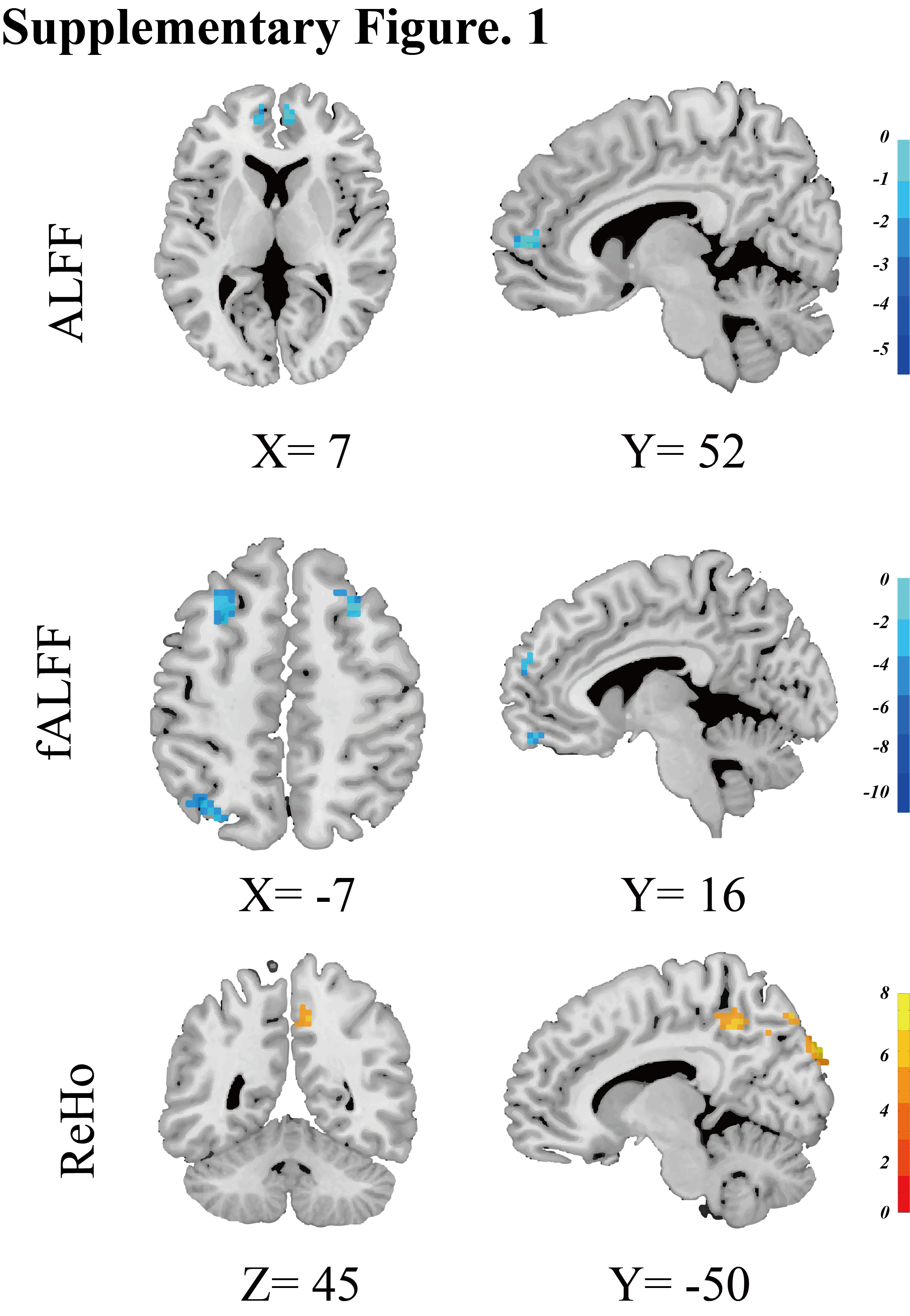


**Legends for Supplementary Figure. 1：**

Supplementary Figure. 1 shows the chronic insomnia group show lower ALFF and fALFF in prefrontal regions and higher ReHo in the Precuneus. **Abbreviations:** ALF, Amplitude of Low frequency fluctuation; fALFF, Fractional ALFF; ReHo, Regional homogeneity.
